# Anxiety predicts math achievement in kindergarten children

**DOI:** 10.3389/fpsyg.2024.1335952

**Published:** 2024-02-27

**Authors:** Bernadett Svraka, Carolina Álvarez, Dénes Szücs

**Affiliations:** ^1^Department of Education, Faculty of Primary and Pre-School Education, ELTE, Eötvös Loránd University, Budapest, Hungary; ^2^Doctoral School of Mental Health Sciences, Semmelweis University, Budapest, Hungary; ^3^MTA-SZTE Metacognition Research Group, Szeged, Hungary; ^4^National Laboratory for Social Innovation, Budapest, Hungary; ^5^Centre for Neuroscience in Education, Department of Psychology, University of Cambridge, Cambridge, United Kingdom

**Keywords:** math, general anxiety, sex, socioeconomic status, kindergarten, preschool

## Abstract

**Introduction:**

Math anxiety (MA) is an academic anxiety about learning, doing, and evaluating mathematics, usually studied in school populations and adults. However, MA likely has its origins before children go to school. For example, studies have shown that general anxiety (GA) for everyday events is less separable from MA in primary than in early secondary school. This suggests that GA may be a precursor of MA. For this reason, here, we have examined whether GA is already associated with math achievement at the end of kindergarten.

**Methods:**

We tested 488 Hungarian kindergarten children aged 5.7 to 6.9 years (55% girls) and analyzed the effect of GA, sex, and family SES on math achievement in kindergarten children.

**Results:**

Strikingly, confirming results from primary school children, we found that GA negatively correlated with math achievement already in this preschool population. Higher GA levels had a stronger negative effect on girls’ than boys’ math achievement. However, there were no significant sex differences in math achievement in kindergarten. Additionally, family socioeconomic status was the strongest predictor of math achievement.

**Discussion:**

We speculate that high GA in preschool is a plausible early precursor of later high MA. Early interventions could aim to control GA levels before children start formal schooling.

## 1 Introduction

Math anxiety (MA) is an academic anxiety about learning and doing mathematics (Ashcraft, 2002). Students with high levels of MA tend to avoid mathematics and math related careers, potentially negatively impacting their career progression and earning potential (Silver et al., 2021). The context of MA is increasingly investigated in primary and secondary school populations (e.g., see Ramirez et al., 2013; Carey et al., 2017; Barroso et al., 2021). However, there is very little data from kindergarten children whereas MA likely has its origins before children go to school (Lu et al., 2021). Notably, general anxiety (GA) for everyday events and MA correlate similarly with math achievement in early primary school while MA becomes a more specific correlate of math achievement during later schooling (Carey et al., 2017). Hence, initially MA and GA may be less separable, and GA may be an important precursor of MA: High MA may develop in children susceptible to high GA (Carey et al., 2017). For this reason, here, we have considered GA in kindergarten as a potential precursor of later school MA and examined GA in relation to math achievement in a sample of kindergarten children in Hungary. Family Socio-Economic Status (SES) and sex were also considered as several studies have shown that they represent important background variables affecting children’s math development and MA (Devine et al., 2012; Carey et al., 2017; Coley et al., 2020; DePascale et al., 2023). Our study can provide information on the early stages of the GA and math achievement relationship.

### 1.1 Anxiety and math achievement

In kindergarten children, math achievement has been generally measured through standardized tests measuring diverse abilities (e.g., math problem solving, calculation, number identification) (Becker et al., 2022 [*N* = 310]; Lu et al., 2021 [*N* = 355]; Ouyang et al., 2022 [*N* = 190]). Teacher reports of children’s math achievement have also been used at times (Romano et al., 2010 [*N* = 1,521]).

To our knowledge, only two studies measured MA directly in kindergarten children. Petronzi et al. (2019) designed the Children’s Mathematics Anxiety Scale UK for children between ages 4–7 years. This scale was validated in 163 children, identifying a single factor model of Online Mathematics Anxiety related to the experience of an entire mathematics lesson, and it was negatively related to math achievement. Lu et al. (2021) designed the Young Children’s MA Scale (YCMAX) and identified two factors of MA in 355 US kindergarten children (exact age was not reported): worry and somatization. Higher worry and somatization were negatively related to math achievement, feelings of competence in math, liking math, and their teachers’ perceptions of interest and need for extra support in math.

MA and math achievement significantly correlated with children’s evaluation of math (how good they thought they were in math and how much they liked math) at the end of first grade (7.5-year-olds); but not between MA and math achievement (Krinzinger et al., 2009 [*N* = 140]). Contrary to these results, a study with first and second graders found a negative relationship between MA and math achievement, with the belief that ability is fixed being a predictor of higher MA (Gunderson et al., 2018 [*N* = 634]). In another study with first and second graders, MA has been negatively related to the use of advanced problem-solving strategies related to math achievement. Notably, children with higher working memory were less likely to use advanced problem-solving strategies when they had high MA, underachieving in math compared to children with lower working memory (Ramirez et al., 2016 [*N* = 564]). MA has also been negatively correlated to secondary students’ math achievement, independently of students’ GA, with girls having higher MA than boys in both primary and early secondary school (Hill et al., 2016 [*N* = 981]). A meta-analysis of 747 effect sizes from research between 1992 and 2018 confirmed the relationship between MA and math achievement in schoolchildren and adults (first grade to graduate students and nonstudent adult samples) (*r* = −0.28), finding five significant moderators: grade level (first and second grade: *r* = −0.26, third and fifth grade: *r* = −0.20, sixth to eighth grade: *r* = −0.30, ninth to twelfth grade: *r* = −0.34, postsecondary: *r* = −0.24, and non-student adults: *r* = −0.32), math ability, adolescent/adult MA scales, math topic of GA scale, and math assessments (Barroso et al., 2021). This meta-analysis did not find studies with kindergarten children, as MA has usually been studied in older children and adults.

GA in kindergarten has usually been measured through parental report (Hastings et al., 2015 [*N* = 375]; Heberle and Carter, 2020 [*N* = 94]; Herrmann et al., 2018 [*N* = 1,093]) but some studies used teacher reports (Guhn et al., 2020 [*N* = 134,094]), semi-structured interviews (Ezpeleta et al., 2014 [*N* = 622]), or physician-assigned ICD-9 or ICD-10 (International Classification of Diseases) diagnostic codes used in administrative healthcare records (Guhn et al., 2020). A longitudinal study found that GA (maternal report) in junior or senior kindergarten children (4-or 5-year-olds, respectively) (*M* age = 4.8 years old) predicted math achievement (test score) in third grade. In turn, math achievement (teacher report) in junior or senior kindergarten predicted GA (maternal report) in third grade (Romano et al., 2010). Cross-sectional studies supported these findings. Szczygieł (2020a) [*N* = 369] found a weak negative relationship between GA (child report) and math achievement in second grade, although not in first grade.

MA and GA may be intertwined: in third and fifth graders (8 to 12 years old), children’s ratings of their trait GA (i.e., their typical level of GA) was weakly negatively related to math achievement (tests and teacher ratings) but strongly (*r* = 0.46) related to MA (Justicia-Galiano et al., 2017 [*N* = 167]). Also, Hill et al. (2016) reported moderate to strong (0.36 < *r* < 0.47) correlations between MA and GA in third to fifth grade children. Crucially, Carey et al. (2017) [*N* = 1,746] found that MA and GA correlated similarly with math achievement in third grade children, but the correlation of MA and math achievement became much more specific by seventh/eighth grade. The authors suggested that GA may be a precursor of MA: Early high GA may predispose some (but not all) children to have high MA later.

Some studies have differentiated between state anxiety (how a person feels at a particular moment) and trait anxiety (how they generally feel) in children (Orbach et al., 2019 [*N* = 1,179]) and in adults (Daches Cohen et al., 2021 [*N* = 117]). Trait math anxiety refers to an acquired disposition to perceive a variety of math-related situations as threatening and is measured with self-report questionnaires about hypothetical or retrospective math-related situations. On the other hand, state math anxiety is a temporary anxious response to the current math-related situation and involve arousal of the autonomic nervous system and is measured with real-time self-reports about their present experience (Orbach et al., 2019; Daches Cohen et al., 2021). In fourth and fifth grade children, there was a negative correlation between state MA and math achievement, but not between trait MA and math achievement (Orbach et al., 2019, 2020 [*N* = 646]). Considering these definitions, in the present study we focus on children’s anxiety as a trait.

### 1.2 Sex differences

There is mixed evidence regarding sex and math achievement. Some studies suggest no significant sex differences in math achievement in pre-kindergarten (Barbarin et al., 2006 [*N* = 501]; Becker et al., 2022) and kindergarten children (Matthews et al., 2009 [*N* = 268]), kindergarten to third grade children (Susperreguy et al., 2022 [*N* = 367]), first and second grade children (Szczygieł, 2020b), and longitudinally from kindergarten and first grade to third and fifth grade (Herbert and Stipek, 2005 [*N* = 345]). However, there is also evidence of sex differences in math achievement, with girls having higher math achievement in kindergarten and boys in first grade (Fischer and Thierry, 2022 [*N* = 2,633]), or higher achievement in boys between kindergarten and fifth grade (Li-Grining et al., 2010 [*N* = 10,666]). Some studies have found no sex differences in math achievement during the first 2 years of primary school (Szczygieł, 2020a) or across four time points (kindergarten, first, third, and fifth grade) (Herbert and Stipek, 2005). Other studies have found sex differences favoring girls’ math achievement in kindergarten (*M* age = 4.8 years old) and favoring boys in first grade (*M* age = 6.6. years old) (Fischer and Thierry, 2022). Another study has found that boys had higher math achievement growth rates between kindergarten and fifth grade in comparison to girls (Li-Grining et al., 2010).

There are also mixed findings regarding sex differences in anxiety: Higher MA was reported in second and fourth grade girls (Van Mier et al., 2019 [*N* = 124]) and in 9-year-olds girls (Devine et al., 2018 [*N* = 1,757]). Higher MA and GA were reported in third to fifth grade girls (Hill et al., 2016) and higher test, MA, and GA in fourth grade girls (Carey et al., 2017). Szczygieł (2020a) also found higher GA in first and second grade Polish girls than boys. Additionally, GA mediated the relationship between sex and MA; therefore, sex affected the differences in GA, which in turn affected MA. In 5-to 12.9-year-old children, sex was related to the first episode of GA, with girls having higher rates of GA disorders (Essau et al., 2018 [*N* = 816]). However, an earlier meta-analytic review of 555 effect sizes from 166 studies of 0 to 17-year-olds found no significant sex differences in GA (Chaplin and Aldao, 2013 [*N* = 21,709]). Some studies reported no sex differences in GA in 4-to 8-year-old children (Paulus et al., 2015 [*N* = 1,342]) and in MA in first and second grade (Ramirez et al., 2013 [*N* = 154]).

### 1.3 Environmental factors

Parental and teacher MA have been linked to children’s MA and math achievement in school populations (Beilock et al., 2010 [*N* = 114 children, 17 teachers]; Maloney et al., 2015 [*N* = 379 children, 133 parents, 76 teachers]; Soni and Kumari, 2017 [*N* = 595 parent–child dyads]; Szczygieł, 2020a [*N* = 241 children, 227 parents, 30 teachers]). In preschool populations, findings are mixed. Four-year-old children tended to have higher math achievement when their parents believed that math skills were important before starting kindergarten. Parents who believed that math skills before starting kindergarten were important had children with higher math achievement and, perhaps surprisingly, the effect was stronger when parents had high MA (Silver et al., 2021 [*N* = 114]). There are also findings regarding a positive relationship between the home numeracy environment and four-year-old children’s math skills when parents had lower math anxiety (Cosso et al., 2023 [*N* = 121 parent–child dyads]). In contrast, another study found parents’ MA to be negatively related to change in five-year-old children’s math achievement within the year (controlling for math achievement at the beginning of the study and demographics), showing no sex differences (Becker et al., 2022). Preschool teachers’ MA has been negatively related to their self-assessment of math abilities (Geist, 2015 [*N* = 31]). Teachers who felt confident about their math abilities and knowledge also liked math, thought that math was important in preschool, planned to teach math in their classroom, and used age-appropriate methods to do so.

Socioeconomic status has been related to math achievement at pre-kindergarten entry in four (Barbarin et al., 2006) and 5-year-old kindergartners (DePascale et al., 2023 [*N* = 403]; Galindo and Sonnenschein, 2015 [*N* = 19,280]). Lower SES children had less sophisticated math solution strategies (DePascale et al., 2023). Another study found that math proficiency at kindergarten entry (proficient or limited proficient math skills) and the home learning environment, especially access to learning tools (e.g., number of books), mediated the negative relation between SES and kindergarten math achievement. Lower SES children proficient in math at kindergarten entry in a more enriched home learning environment developed higher math achievement at the end of kindergarten (Galindo and Sonnenschein, 2015). Abry et al. (2015) [*N* = 2,650] defined “misalignment” as the difference between preschool and kindergarten teacher’s ratings of the most important early (academic, self-regulatory, and interpersonal) school competences for children entering kindergarten. Larger misalignment about all three types of school competences (preschool teachers rated each competence higher than kindergarten teachers did) was associated with lower math achievement in kindergarten, and this effect was stronger in low SES children. A cross-sectional study, including children from kindergarten to third grade, found a math achievement gap between children from low and high SES. This gap increased with time, especially in third grade (Susperreguy et al., 2022). A longitudinal study found that the kindergarten entry SES gap in math achievement grew during the summer months, likely because high SES parents provided more out-of-home summer enrichment activities (e.g., going to a library, museum, or zoo). Between kindergarten and second grade, there was a 25% growth in the entry SES gap in math achievement (Coley et al., 2020 [*N* = 4,000]). Additionally, SES has been negatively related to children’s internalizing problems (a maternal report measure including GA) 4 years later, when children were 6.4 to 11.7 years old (Hastings et al., 2015).

Lower SES has been related to higher mother-reported GA of their three-year-old children (Ezpeleta et al., 2014), in kindergarten children (teacher and physician reports) (*M* age = 5.7) (Guhn et al., 2020), and in a group of 2.5 to 12-year-old children (parent report of emotional problems, including GA) (Herrmann et al., 2018). Children’s GA (parent report) has been related to children’s perceived financial disadvantage (having enough money and as much money as other parents) and negatively related to a family socioeconomic advantage indicator (felt poverty, income, social exclusion, felt support, education, and employment) in a group of 4-to-9-year-olds (Heberle and Carter, 2020). Contrary to these results, a study of 6-year-old children found no significant differences across SES (measured as ownership of consumer durable goods) for GA (maternal report) (Petresco et al., 2014 [*N* = 3,585]).

### 1.4 The present study

Here, we tested typically developing kindergarten children. Our main research question was whether GA, SES, and sex were predictors of mathematics achievement in Hungarian kindergarten children. A secondary question was whether there were differences in GA depending on SES and sex.

First, we expected a significant relationship between SES and GA, as most previous studies found in children from 2.5 to 11.9 years of age (Ezpeleta et al., 2014; Hastings et al., 2015; Herrmann et al., 2018; Guhn et al., 2020).

Second, as described above, some studies reported sex differences in GA in kindergarten (Hill et al., 2016; Carey et al., 2017; Essau et al., 2018; Szczygieł, 2020b) while others have not found such differences (Chaplin and Aldao, 2013; Paulus et al., 2015). Hence, we examined sex differences in GA.

Third, in line with the observations that MA does not yet clearly separate from GA in first grade (Hill et al., 2016) and fourth grade (Carey et al., 2017) school children, we expected that GA would be related to mathematics achievement in kindergarten children.

Fourth, we expected SES to correlate with math achievement, in line with earlier evidence from preschool to primary school years (Barbarin et al., 2006; Galindo and Sonnenschein, 2015; Coley et al., 2020; Susperreguy et al., 2022; DePascale et al., 2023).

Finally, we examined whether there were sex differences in math achievement.

## 2 Method

### 2.1 Participants

We report data from 488 typically developing children in Hungary (54.5% girls, 45.5% boys; M age = 6.34 years; SD = 0.36; range = 5.75 to 6.92 years). Children took part in the assessment procedures of the Pedagogical Service (see below). The sample size was chosen to be large enough to be able to detect small effect size correlations and group differences robustly but as many children were sampled as possible (achieving 0.9 power to detect *r* = 0.2, two-tailed at *α* = 0.05 requires a sample size of 255; achieving power 0.9 to detect a *D* = 0.2 effect size with two-tailed *t*-tests at *α* = 0.05 requires a sample size of 216; G-Power 3.1.9.4; Faul et al., 2009). 13% of the children came from disadvantaged families (entitled to child support benefit). Based on Hungarian law, a child is considered to come from a disadvantaged family when the parents’ highest level of education is primary school, a parent had been registered as a job seeker for at least 12 months, or the family’s living conditions are inadequate for healthy child development (VIII/67/A § of the XXXI / 1997 act (1) on the protection of children and guardianship administration).

To provide further background, in Hungary preschool is a professionally independent educational institution of the public education system and a supplement to family education. It is provided free of charge from age three, or slightly earlier, if the child is deemed ready to start it, until the child enters school (normally at 6 years of age). The law states that children who turn 6 by 31 August must go to school, therefore some children start school by the age of 6 and others closer to age 7. The pedagogical activity system and material environment of preschool ensure the most suitable conditions for the development and education of preschool children. Preschool aims to provide optimal social and personality development for transitioning to elementary school. The goal of preschool education is to promote the versatile and harmonious development of preschoolers, the emergence of the child’s personality, and the reduction of disadvantages, considering age and individual characteristics and different developmental rates (Hungarian Government, 2012). Regarding mathematics knowledge, by 5–6 years of age children should be familiar with numbers 1 to 10, should be able to count up or down in sequence, should know which quantity is smaller or larger than others, which sets have more or less, and how to make sets equal. Children may learn to perform simple operations like 3 + 1 or 4–1. Finger counting can be encouraged.

### 2.2 Ethics statement

Ethical approval was granted by the Research Ethics Committee of the Faculty of Preschool and Primary Education, Eötvös Loránd University, Budapest (ELTE) (Number: KE2022/004).

### 2.3 Procedure

Data collection was carried out in the Pedagogical Service, an institution where psychological and educational assessments of students are conducted. All children who are about to start school need to be assessed. Parents were invited to participate in the study and those who agreed signed a General Data Protection Regulation consent form allowing data to be used anonymously for research purposes. Children were invited to participate and only three children declined and therefore were not assessed. Data collection was supervised by special education teachers, with the assistance of undergraduate and master’s students of ELTE. Children were tested individually. Researchers worked in pairs, one administering the test and the other observing. Parents could be in the room while the child was being tested and they completed parental questionnaires afterwards. Test scores were calculated by special education teachers.

### 2.4 Measures

#### 2.4.1 Math achievement

Dyscalculia Pedagogical Examination (DPV) (Dékány et al., 2020). DPV is a test regularly used by the Hungarian Pedagogical Service and it measures the development of cognitive functions and skills (e.g., understanding of numbers and operational concepts). This test is used for assessing the arithmetic abilities of children in general, for mapping sub-skill weaknesses underlying difficulties in learning basic arithmetic skills and for screening out mathematical underachievement (dyscalculia).

DPV assesses orientation, counting, mechanical counting, invariance, number memory, global quantity recognition, number name vs. quantity matching, quantitative relations, basic concepts, operations in action, series-analogies, number symbols, and word problems.

Higher DPV scores indicate better skills. Children can be classified into three categories: without difficulties, milder difficulties, and math learning disorder. The possible range of scores was 0–267. Cronbach’s alpha with this sample was 0.86.

#### 2.4.2 General anxiety

The Hungarian translation of the Child Behavior Check List (CBCL) was used, an instrument to evaluate children’s behavioral and emotional problems (Achenbach and Rescorla, 2000; Achenbach and Rescorla, 2001). This test has been adapted for the Hungarian population with good to high reliability scores (Cronbach’s alpha for the Anxious/Depressed scale was 0.81 and for the Somatic Complaints scale 0.66) (Rózsa and Kő, 2015).

Parents answered two subscales: Anxious/Depressed (e.g., the child is overly fearful and anxious) and Somatic complaints (e.g., The child has stomachache or abdominal cramps for no known medical reasons). Each item is answered as belonging to one of the following categories: (i) not true, (ii) somewhat or sometimes true, and (iii) often true or very true. The corresponding score of each category is 0, 1 and 2 points, respectively. The total raw score of each scale was calculated by adding the score of all corresponding items. Mothers answered the CBCL in 356 cases, both parents answered the CBCL in 98 cases, and fathers answered the CBCL in 34 cases. The possible range of scores on the Anxious/Depressed subscale was 0–15 and on the Somatic complaints subscale 0–11.

Here, GA was measured by the average value of the Anxious/Depressed and Somatic complaints subscales (*r* = 0.72).

### 2.5 Data analysis

We conducted descriptive statistics and frequency analysis for the main variables. We conducted Spearman’s correlations to examine associations between math achievement, GA, SES, sex, and age. We checked for multicollinearity in these variables by computing the Variance Inflation Factor (VIF). We performed a two-way ANOVA to analyze the effect of SES and sex on GA. We ran multivariate regression analysis to test the contribution of GA, SES, sex, and age on math achievement. We ran analyses in R version 4.0.4 (2021-02-15) (R Core Team, 2021).

## 3 Results

### 3.1 Descriptive statistics

Descriptive statistics are shown in Table 1.

**Table 1:** Descriptive statistics of main measures.

	All (*N* = 488)		Girls (*N* = 266)		Boys (*N* = 222)		High SES (*N* = 424)		Low SES (*N* = 64)	
Variables	*M*	*SE*	*M*	*SE*	*M*	*SE*	*M*	*SE*	*M*	*SE*
Math achievement	10.3	0.1	10.5	0.2	10.1	0.2	10.9	0.1	6.8	0.5
GA	1.3	0.1	1.5	0.2	1.2	0.2	1.0	0.1	4	0.5

### 3.2 Zero-order correlations

Table 2 shows zero-order correlations for all variables. We found strong correlations between math achievement and GA and SES. We also found strong correlations between GA and SES. We found no significant correlations between sex and math achievement, GA, and SES.

**Table 2:** Zero-order correlations.

	1	2	3	4	5
1. Math achievement	1				
2. GA	−0.53***	1			
3. SES (disadvantaged)	−0.43***	0.39***	1		
4. Sex (girls)	0.05	0.02	−0.08	1	
5. Age	−0.03	−0.15**	0.01	−0.07	1

*p* < 0.05 (*), *p* < 0.01 (**), *p* < 0.001 (***). SES: 0 = not disadvantaged, 1 = disadvantaged. Sex: 1 = boys, 2 = girls.

### 3.3 Variance inflation factor (VIF)

We checked the VIF with GA, sex, and SES. VIF values were below 1.2. This indicates that predictor variables were not multi-collinear.

### 3.4 ANOVA

To answer our secondary question (are there differences in GA depending on SES and sex?), we performed a two-way ANOVA analyzing the effect of SES and sex on GA. Girls had marginally higher scores in the overall GA scale (sex main effect: *F* = 3.83, *df* = 1,484, *p* = 0.051). Low SES children had higher scores on the overall GA scale: *F* = 82.99, *df* = 1,484, *p* < 0.001. There was no SES by sex interaction.

### 3.5 Regressions

To answer our main question (are GA, SES, and sex predictors of mathematics achievement in Hungarian kindergarten children?), we fitted our main model predicting math achievement on normalized data. Beta values are shown in Table 3. This model explained 34% of the variance (*R*^2^ = 0.34, *F*(6, 481) = 42.61, *p* < 0.001).

**Table 3:** Standardized regression coefficients for main and interaction effects.

Variable	*β*	*p*	95% CI
GA	−0.37	<0.001	[−0.46, −0.29]
Sex	0.06	0.097	[−0.01, 0.13]
SES	−0.34	<0.001	[−0.43, −0.25]
GA: SES	0.05	0.274	[−0.01, 0.11]
GA: Sex	−0.12	0.003	[−0.20, −0.04]
SES: Sex	0.07	0.112	[−0.01, 0.14]

**Figure 1:**
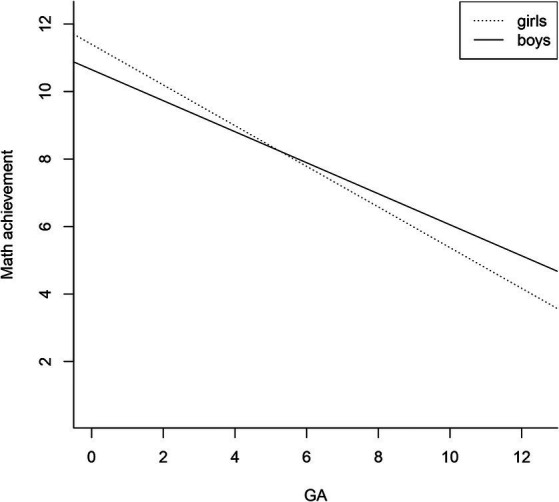
Interaction between GA and sex on math achievement. This figure shows an interaction effect of GA and sex on math achievement. Higher levels of GA have a stronger effect on girls’ levels of math achievement. Axes show standardized measurement units.

We also built a stepwise regression model using the single inbuilt R function “step.” This function initially includes all predictors in the model and then omits irrelevant predictors. Beta values are shown in Table 4. In line with the outcomes of our main model this procedure only removed the non-significant GA: SES interaction term from the initial model. The final model also explained 34% of the variance (*R*^2^ = 0.34, *F*(5, 482) = 50.87, *p* < 0.001). As seen in Figure 1, the GA by sex interaction appeared because girls with low anxiety had higher math achievement than boys whereas girls with high anxiety had lower math achievement than boys.

**Table 4:** Standardized regression coefficients for main and interaction effects.

Variable	*β*	*p*	95% CI
GA	−0.36	<0.001	[−0.43, −0.28]
Sex	0.06	0.110	[−0.01, 0.13]
SES	−0.32	<0.001	[−0.40, −0.24]
GA: Sex	−0.13	0.002	[−0.21, −0.05]
SES: Sex	0.07	0.088	[−0.01, 0.15]

## 4 Discussion

MA is a stable correlate of math achievement in schoolchildren, and it likely has its origins before children go to school. GA for everyday events may serve as an important precursor of MA (Hill et al., 2016; Carey et al., 2017). However, little is known about the math achievement and GA relationship in kindergarten (Romano et al., 2010; Galindo and Sonnenschein, 2015; Fischer and Thierry, 2022). To fill this research gap, here we have analyzed GA, sex, and family SES as predictors of math achievement in kindergarten children. Additionally, we also analyzed relationships between predictor variables (i.e., differences in GA depending on SES and sex).

### 4.1 Family SES and children’s GA

As expected, we found a strong relationship between SES and GA (*r* = 0.4): children from disadvantaged families experienced higher levels of GA. This effect size is stronger than the one reported by a study with eight-year-old children, where children from lower SES presented higher GA (*r* absolute value = 0.12, Hastings et al., 2015). This discrepancy may also be due to SES measurement differences. Unlike Hastings et al. (2015), in our sample the definition of disadvantage was based on low parental education level, job seeking for more than a year, or insufficient living conditions. Public policies aimed at children from disadvantaged backgrounds could prioritize the above three aspects. For example, promoting students to complete high school education and have access to higher education, assisting job candidates in their application process, and social support enabling families’ minimum conditions to raise their children. Aiming at poverty reduction as a public health issue has also been raised by previous research studying the impact of social and economic factors on children’s mental health (Guhn et al., 2020).

### 4.2 Children’s sex and GA

Girls had slightly higher scores on GA than boys (1.5 ± 0.2 vs. 1.2 ± 0.2). Future studies could use longitudinal design to corroborate the age when girls start showing higher GA than boys. Current evidence in young children is mixed: no sex differences in GA have been found in 4-to-8-year-old children (Paulus et al., 2015) but studies with first and second grade children have found higher GA in girls than boys (Hill et al., 2016; Szczygieł, 2020a).

### 4.3 Children’s GA and math achievement

We found a strong negative relationship (*r* = −0.53) between GA and math achievement: On average, children experiencing higher levels of GA had lower math achievement than children with lower GA. Previous studies reported less strong GA and math achievement correlations than the one found here (*r* = −0.16, Justicia-Galiano et al., 2017; *r* = −0.17, Szczygieł, 2020a). However, earlier studies tested Grade 1 to 5 children with self-report questionnaires rather than referring to maternal reports about kindergarten children as we did here. Studies with school-age children found that parents report more GA symptoms than children (Bowers et al., 2020 [*N* = 360]; Cole et al., 2000 [*N* = 562]; Nauta et al., 2004 [*N* = 484 parent–child dyads, 261 control group]; Weems et al., 2010 [*N* = 202]). Perhaps primary school children do not report as many GA symptoms as mothers observe and gathering information from multiple reports (e.g., from parental, teacher, and child self-reports) could shed light on relevant differences. Differences between child and parent report of anxiety could be due to social desirability, parent–child communication about inner thoughts and feelings, the context, and focus on different aspects of the symptomatology (Cole et al., 2000; Nauta et al., 2004; Weems et al., 2010; Bowers et al., 2020).

Future longitudinal studies could analyze the direction of causality between GA and math achievement in kindergarten (for review see Carey et al., 2016). First, children who experience high levels of GA and low math achievement might need help coping with feelings of worry, insecurity, and fear. Teachers or clinicians could identify whether these feelings are related to the classroom context or respond to other stressors. Second, children whose math achievement is low might become anxious as they might be under pressure to perform, might perceive differences with their peers, and may feel insecure of their own abilities to improve. There is evidence that 5-to 12-year-old children’s growth or fixed mindsets about intelligence (a trait you cannot change vs. a quality you can develop) influences their motivation and achievement (Haimovitz and Dweck, 2017). Children benefit from growth mindsets, and therefore teachers and parents could promote focusing on their learning process (the role of effort, effective strategies, and guidance) and progress over time. In a role-play experiment, 5-to 6-year-olds received either person-or process-focused critical feedback from a teacher character after a mistake. Children who received person-focused criticism endorsed a fixed mindset and were less resilient to future mistakes; children who received process-focused criticism endorsed a growth mindset and were more resilient at coping (Kamins and Dweck, 1999 [*N* = 67 study 1, 64 study 2]). GA and math achievement relationships could be bidirectional and reinforce each other over time, with high GA negatively impacting math achievement, and lower achievement or feelings of insecurity about a subject, increasing feelings of GA (Carey et al., 2016). In this case, both intervention types should be integrated to effectively support children. Further, GA may interact with early low math achievement, which might lead to MA and lower math achievement. Therefore, early support for children struggling in their first experiences of math may also prevent the appearance of MA later on.

Another potential explanation for the effect of GA on math is offered by the Attentional Control Theory (Eysenck et al., 2007). This theory proposes that anxiety decreases the functioning of the goal-directed attentional system (attentional control) and increases the influence of the stimulus-driven attentional system (e.g., threat stimuli). Two executive functions are involved in attentional control: inhibition control (inhibiting attention to task-irrelevant stimuli) and task shifting (switching attention between and within tasks). Anxiety therefore would lead to higher distractibility. However, quality of performance may not be affected by anxiety when compensatory strategies are used. Future studies could add a measure of executive functions to test its role in the relationship between GA and math achievement in young children. The evidence for math anxiety shows that inhibition control plays an important role. In fourth and fifth grade children, experiencing high state-MA impaired their inhibition control processes and their math achievement was negatively affected (Orbach et al., 2020, *N* = 646). In adults, math anxiety has been found to impair cognitive systems related to inhibition control, therefore leading to higher distractibility when performing math tasks (Pletzer et al., 2015 [*N* = 36]; Van Den Bussche et al., 2020 [*N* = 92]). Future studies should further investigate whether inhibition control has a similar effect in preschool children.

### 4.4 Family SES and children’s math achievement

As hypothesized, we found a strong negative relationship between SES and math achievement (*r* = −0.43): children from disadvantaged households had lower math achievement than their more affluent peers. We found a stronger correlation than previous studies with five-year-old children (0.23 < *r* < 0.36) (Coley et al., 2020; Susperreguy et al., 2022; DePascale et al., 2023). This difference may be due to the type of measurement used in this study, where SES was a dichotomous variable indicating the presence of disadvantage, in comparison with measures of school SES or composite measures of household income and parental education and job prestige. Our results suggest that the math achievement of children with socioeconomic disadvantage is negatively affected. This highlights the need for additional academic support already during kindergarten. As the SES gap in math achievement tends to increase with age (Susperreguy et al., 2022), interventions in kindergarten have the potential to promote equity of opportunities from a young age. For example, the Hungarian preschool system, where the study was done, aims to alleviate differences in SES from age 3 from which preschool is obligatory. Hence, this issue is even more pressing in countries which may not have a similar institutional support system.

A mechanism that could be investigated is the role of the home numeracy environment. Parents with higher education levels engage in math activities with their children more often, and this relationship is stronger for younger children (four-year-olds and kindergarteners) (Leyva et al., 2021 [*N* = 78]; Susperreguy et al., 2022). As parents promote more math activities, children have more opportunities to practice and develop their skills before starting formal schooling. Preschools working with children from lower SES backgrounds could focus on addressing this issue, providing additional activities that foster children’s math development and guiding parents on concrete ways to practice math at home (Galindo and Sonnenschein, 2015).

### 4.5 Children’s sex and math achievement

We found no significant difference in math achievement between girls and boys, similar to previous studies with slightly lower sample sizes (268 < *N* < 369; Herbert and Stipek, 2005; Matthews et al., 2009; Szczygieł, 2020b). In addition, both girls and boys saw their math achievement affected when experiencing GA. However, there was also a significant interaction effect of GA and sex on math achievement: higher GA levels had a stronger negative effect on girls’ than boys’ math achievement. We speculate that the interaction effect may indicate that GA indeed mediates sex differences in MA in primary school (Carey et al., 2017; Szczygieł, 2020a). Speculatively, the early sex discrepancy shown here may also be a precursor of often lower math self-concept in girls than boys. For example, even fourth grade girls have worse math self-concept than boys irrespective of their performance level according to data from the Trends in International Mathematics and Science Study (TIMSS) 2015 (Mejía-Rodríguez et al., 2021 [*N* = 32 countries]).

A key question is why girls’ math achievement is more affected than boys’ at high levels of GA and whether this effect is specific to math or rather, it extends to other academic subjects. If the effect is specific to math, a potential causal factor may be the implicit or explicit influence of parents’ and teachers’ math gender stereotypes (Beilock et al., 2010; Becker et al., 2022) whose impact can already be shown in 6-year-old kindergarten children (del Río and Strasser, 2013 [*N* = 81]). For a causal test of this possibility, it remains to be seen whether gender-stereotype targeting interventions can alleviate the greater impact of GA in girls than in boys in kindergarten. For example, for 5-to 7-year-olds, mothers’ rejection of math gender stereotypes is a protective factor for girls’ math, whereas parental endorsement of math gender stereotypes is a risk factor for girls’ math (del Río et al., 2019 [*N* = 180]; Tomasetto et al., 2011 [*N* = 124]).

### 4.6 Limitations and future studies

The cross-sectional design of our study does not allow for causal explanations. Longitudinal studies can provide more detailed information on children’s trajectories of math achievement development in relation to GA, sex, and SES. Besides, we do not hold parental information beyond SES and no information about teachers. Recent studies have shown that maternal education was the best predictor of kindergarten children’s math achievement and not parental MA or achievement (Bellon et al., 2022 [*N* = 83 mother–father-child triads]). However, the sample was relatively small, so generalization requires caution. Another study found that child math achievement was negatively related to their MA when parents had low MA, but there was no relation between child math achievement and MA when parents had high MA and interacted frequently with their children in home numeracy activities (e.g., playing cards). The authors suggested that the involvement of high MA parents was beneficial to their children at this age, as they may be more aware of their children’s MA (Guzmán et al., 2023 [*N* = 311]). Future studies could include parental MA to gain a better understanding of its role for children who are starting to learn math. Future studies would also benefit by considering parents’ and teacher’s measures of gender stereotypes about math. Additionally, family history of learning disabilities (e.g., dyscalculia) or ADHD could be included. Future studies could also use an additional dependent variable besides math achievement to determine the specificity of effects to math.

Few studies have studied spatial anxiety, feelings of nervousness related to carrying out spatial tasks (e.g., mental rotation) (Ramirez et al., 2012), in relation to math achievement in young children. Ouyang et al. (2022) [*N* = 190] and Wong (2017) [*N* = 182] found that spatial anxiety moderated the relationship between spatial skills and math achievement in preschool, and Tian et al. (2022) [*N* = 490] found that spatial skills but not spatial anxiety, mediated sex differences in number line estimation in a group of kindergarten to fourth grade children. Future studies could make comparisons between spatial anxiety, math anxiety, and general anxiety as predictors of math achievement in kindergarten. However, running many more tests with kindergarten children is obviously challenging.

### 4.7 Conclusion

We analyzed the effect of GA, sex, and family SES on math achievement in kindergarten children. We found a strong negative correlation between GA and math achievement. This relationship may be a precursor of the increasingly more specific MA and math achievement relationship in school. Girls with high GA performed worse than boys with high GA. This sex discrepancy may be a precursor of the often-reported higher GA and MA in girls than in boys in school populations. SES was the strongest predictor of math achievement. We suggest working closely with preschool teachers and parents to identify early signs of high GA as it may negatively affect educational outcomes in the long run, especially by facilitating the appearance of academic anxieties, like MA.

## Data availability statement

The raw data supporting the conclusions of this article will be made available by the authors, without undue reservation.

## Ethics statement

The studies involving humans were approved by the Research Ethics Committee of the Faculty of Preschool and Primary Education, Eötvös Loránd University, Budapest (ELTE) (Number: KE2022/004). The studies were conducted in accordance with the local legislation and institutional requirements. Written informed consent for participation in this study was provided by the participants’ legal guardians/next of kin.

## Author contributions

BS: Conceptualization, Funding acquisition, Investigation, Methodology, Project administration, Writing – review & editing. CÁ: Formal analysis, Visualization, Writing – original draft, Writing – review & editing. DS: Conceptualization, Data curation, Formal analysis, Methodology, Writing – review & editing.
